# Gold nanochannels oxidation by confined water†

**DOI:** 10.1039/d0ra05830k

**Published:** 2020-10-07

**Authors:** André M. Batista, Thiago B. de Queiroz, Renato A. Antunes, Alexandre J. C. Lanfredi, Adriano R. V. Benvenho, Jean J. Bonvent, Herculano Martinho

**Affiliations:** Universidade Federal do ABC Av. dos Estados 5001 Santo André-SP 09210-580 Brazil herculano.martinho@ufabc.edu.br +55 (11) 4996 0196

## Abstract

Confined and interstitial water has a key role in several chemical, physical and biological processes. It is remarkable that many aspects of water behavior in this regime (*e.g.*, chemical reactivity) remain obscure and unaddressed. In particular for gold surfaces, results from simulations indicated that the first wetting layer would present hydrophilic behavior in contrast to the overall hydrophobic character of the bulk water on this surface. In the present work we investigate the properties of confined water on Au 〈111〉 nanochannels. Our findings, based on a large set of morphological, structural and spectroscopic experimental data and *ab initio* computer simulations, strongly support the hypothesis of hydrophilicity of the first wetting layer of the Au 〈111〉 surface. A unique oxidation process was also observed in the nanochannels driven by confined water. Our findings indicated that the oxidation product is Au(OH)_3_. Therefore, the Au surface reactivity against confined water needs to be considered for nanoscopic applications such as, *e.g.*, catalysis in fine chemicals, pharmaceuticals, and the food industry green processes.

## Abstract 2

We investigate the properties of confined water on Au 〈111〉 nanochannels. We report an unique oxidation process was also observed in the nanochannels driven by first wetting layer of the surface.

## Graphical abstract

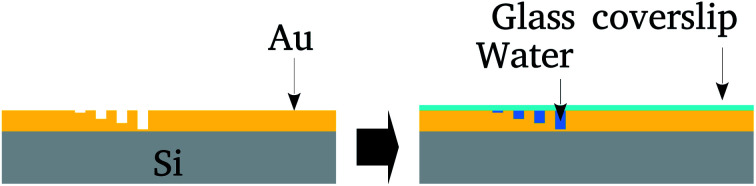

## 1 Introduction

Confined and interfacial water is very important in chemical, physical, and biological processes. However, many of its properties in confinement remain unknown. For this reason, it is relevant to probe how geometric confinements and surface interactions affect the properties of bulk water as well as the substrates containing it.^1,2^

In this sense, a large number of investigations have been performed to study the structure and dynamics of water in diverse systems such as biological environments,^3^ nanoporous silica matrices,^4,5^ vermiculites,^6^ molecular sieves,^7^ and organic coatings.^8^

In particular, some works addressed the issue of corrosion processes and metal reactivity under the action of confined water. Azmat *et al.*^9^ investigated the corrosion of Zn surfaces due to acidified droplets (diameter ∼ 0.1–5 μm). They concluded that the process is dependent on the initial volume of aerosols, oxygen diffusion, surface area to volume ratio and likely the micro-structural features of the underlying metal. Xia *et al.*^10^ investigated the corrosion characteristics of micro- and nano-particles of Cu in distilled water by measuring the absorbance and structure of corrosion products using X-Ray Diffraction (XRD) and Scanning Electron Microscopy (SEM). They concluded that corrosion products of micro-particles increase slowly with increasing immersion time. However, for Cu nano-particles its products increase rapidly with increasing immersion time. Liu *et al.*^11^ investigated the de-hydrogenation on the oxygen-covered surface for Au 〈100〉. They found that the energy barrier of decomposing water strongly reduces the surface of atomic Au covered with oxygen and the O atom can promote dehydration of the H_2_O molecule.

These studies have important technological implications. In fact, Au/oxide catalysts are widely used in important processes, such as partial oxidation of hydrocarbons, hydrogenation of unsaturated hydrocarbons and CO oxidation.^12,13^ Investigations have shown that Au provides catalytically active systems, whereas selectivity and re-usability are advantages over non-catalyzed organic transformations.^14^ Schryer *et al.*^15^ reported that these Au catalysts show increasing activity in the presence of water, unlike other traditional metal oxide catalysts.

In fact, the water:Au surface reactivity at nanoscale is a challenging issue. In the present paper we investigated the oxidation of Au by confined water in nanochannels of variable dimensions produced by nanolithography. The physical–chemical processes occurring in the channels were analyzed by high resolution morphological (atomic force microscopy, scanning electron microscopy), structural (X-ray diffraction, nuclear magnetic resonance), molecular spectroscopy (Fourier-transform infrared absorption, Raman), and elemental analysis (X-ray photoelectron spectroscopy) techniques. *Ab initio* density functional calculations were also performed aiming to furnish possible molecular scenarios to interpret the experimental data.

## 2 Materials and methods

### 2.1 Au metallic thin films

High purity Au (99.99%) was sputtered and deposited on Si substrate (275 μm thick, 〈100〉 orientation, N/P type, from UniversityWafer, Inc.). The Si substrates were cleaned before Au deposition following a three steps sonication washing in: (i) 50/50 solution of Extran detergent and isopropyl alcohol, (ii) isopropyl alcohol, and (iii) deionized water. The washing time in each step was 30 minutes. The deposition was performed on a Leica EM ACE600 sputter coater equipped with quartz crystal for determination of the thin film layer thickness. The thicknesses of produced films were 10, 50, 100, 302 and 600 nm.

### 2.2 Atomic force microscopy (AFM)

The nanolithography and topological characterization was performed on an Agilent AFM/SPM Series 5500 Atomic Force Microscope. Nanolithography was performed under ambient conditions using the vector scanning method. It employed a DLC-coated Si tip (model 190DLC from BudgetSensors Innovative Solutions Bulgaria Ltd.) for static plowing.^16^ The patterns were recorded on a 5 μm^2^ area. The nanochannels topography was imaged using a conventional Si_3_N_4_ tip (in contact mode) that has a low spring constant (about 0.02 N m^−1^), in order to avoid local damage.

**Fig. 1:**
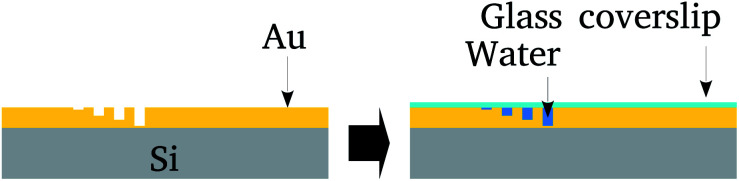
Schematic representation of the nanolithography on the Au film deposited on 275 μm thick N/P Si substrate and water confinement in the channels by spreading a droplet on the surface. After preparation, the Au surface was covered with a glass cover-slip.

Next, the surface was covered by a glass cover-slip and water confined between the cover-slip and nanochannels. After that the cover-slip was removed and samples preserved in a vacuum. Fig. 1 summarizes these steps.

### 2.3 Fourier-transform infrared spectroscopy (FTIR)

The FTIR spectra were collected using an FTIR spectrometer 640-IR FT-IR coupled to a 610-IR microspectrometer (Varian Inc.) with N_2_-cooled Ge detector covering the 600–6000 cm^−1^ spectral window. The detection area was 10 × 10 μm^2^.

### 2.4 Raman spectroscopy

A T64000 Horiba Jobin-Yvon triple Raman spectrometer was used in the subtractive configuration with 1024 × 256 - OPEN-3LD/R CCD detector for Raman scattering measurements. The excitation laser was the Verdi G5 Laser (Coherent Inc.) operating at 532 nm (green) with a power of 1 mW on a 100× objective (laser spot diameter of 1 μm).

### 2.5 Nuclear magnetic resonance (NMR)

The NMR experiments were performed with the sample placed inside a 4 mm Zirconia rotor. The control experiments were performed on a silicone greased Si substrate with the empty rotor as well (*i.e.*, inspecting probe and rotor background). ^1^H spectra and spin–spin relaxation times (*T*_2_) were measured using the spin-echo pulse sequence (*π*/2 − *τ* − *π* − *τ* − acquisition) conducted in a Varian VNMRS 500 MHz spectrometer operating at the resonance frequency of 499.8 MHz, with temperature at 25 °C. The inter-pulse delay (*τ*) of the spin-echo pulse sequence was varied from 67 to 4288 μs and 2048 transients were collected. *T*_2_ was calculated from the single exponential decay fit of the intensity of the deconvoluted and background-subtracted peaks as a function of 2*τ*. The acquisition parameters were *π*/2 pulse length of 2.5 μs and relaxation delay of 5 s. Chemical shifts are reported relative to tetramethylsilane at 0 ppm. The signal was deconvoluted as sum of Lorentz/Gaussian line-shapes. More details are described in the ESI.†

### 2.6 Scanning electron microscopy (SEM)

For morphological characterization, a high-resolution field emission scanning electron microscopy model FESEM JSM-6701F (JEOL) was employed in the secondary electron image mode and low-vacuum scanning electron microscopy SEM (JSM-6010LA, JEOL).

### 2.7 X-Ray diffraction

The crystallographic phase was checked by performing X-ray diffraction experiments at room temperature on a Bruker D-8 Focus diffractometer (Lynseye 1D detector) with Ni-filtered CuK_α1_ radiation in the range from 30° to 60° (0.02° increments) in *θ*–2*θ* configuration.

### 2.8 Density functional calculations (DFT)

#### 2.8.1 Molecular dynamics

All the *Ab initio* molecular dynamics (MD) simulations were performed with the CP2K package.^17,18^ The Gaussian basis set by VandeVondele and Hutter^19^ derived for use with the analytical dual-space pseudo-potentials proposed by Goedecker, Teter, and Hutter (GTH)^20^ was used (cutoff of 250 Ry). These pseudo-potentials were used in conjunction with the Gaussian and plane wave (GPW) scheme^21^ as implemented in the CP2K/QUICKSTEP program. All calculations were performed using the BLYP functional.^22^ The crystallographic structure for Au obtained from refinement of XRD diffraction as reported by Suh *et al.*^23^ was used as the starting point of calculations. A super-cell of Au crystal was hydrated at one side (see Results and discussion section) and then 310 K (Nosé-Hoover thermostat) microcanonical *NVE* ensemble MD ran up to 100 fs.

#### 2.8.2 Vibrational calculations

DFT^17^ was used in order to obtain equilibrium geometries and harmonic frequencies. Calculations were implemented in the CPMD program^24^ using the BLYP functional^22^ augmented with dispersion corrections for the proper description of van der Waals interactions.^22,25^ For all simulations, the cutoff energy was considered up to 100 Ry. The linear response for the values of polarization and polar tensors of each atom in the system was calculated to evaluate the eigenvectors of each vibrational mode.

## 3 Results and discussion

**Fig. 2:**
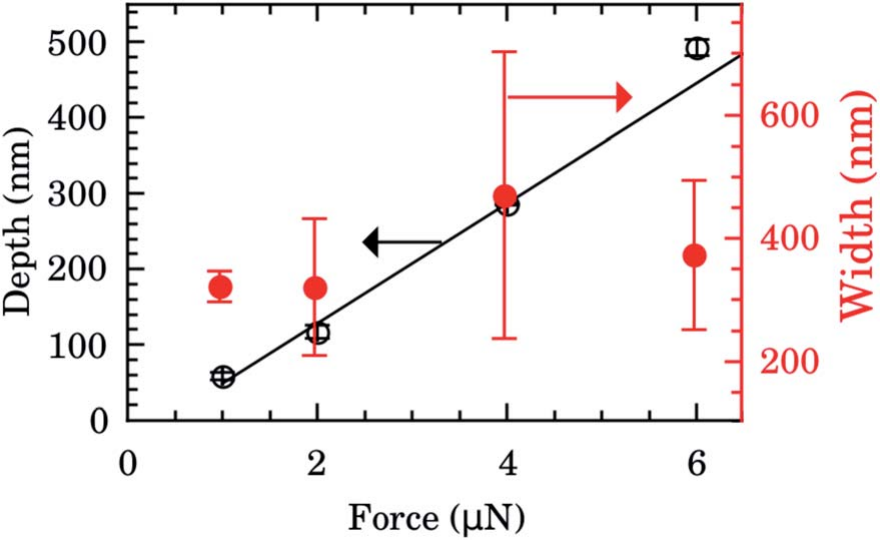
Depth (left scale, black) and width (right scale, red) of nanochannels as function of AFM tip applied force for films with 600 nm of Au. The black line is the best fit to linear model (intercept of −31(6) nm and slope of 80(2) nm μN^−1^).

The depth and width of nanochannels as function of applied AFM tip force are shown in Fig. 2 for films with 600 nm of Au layer. Up to 6 μN, the dependence of depth is almost linear (left scale, black symbols). The best fit of data to the linear model (black line) furnished an intercept of −31(6) nm and slope of 80(2) nm μN^−1^. Controlling the applied force between 1–6 μN we obtained channels with depths of 50–500 nm. Otherwise the values of the width of the nanochannel present a large dispersion (right scale, red symbols) being almost independent of the tip applied force. On average the width was 388(156) nm. Typical 2D AFM microscopy images before and after patterning for the 600 nm Au layer sample are presented in the ESI.† We also checked the DLC-coated cantilever profile by SEM and we notice that the DLC film remained intact after the patterning process. Otherwise the homogeneity of channels would be compromised.

**Fig. 3:**
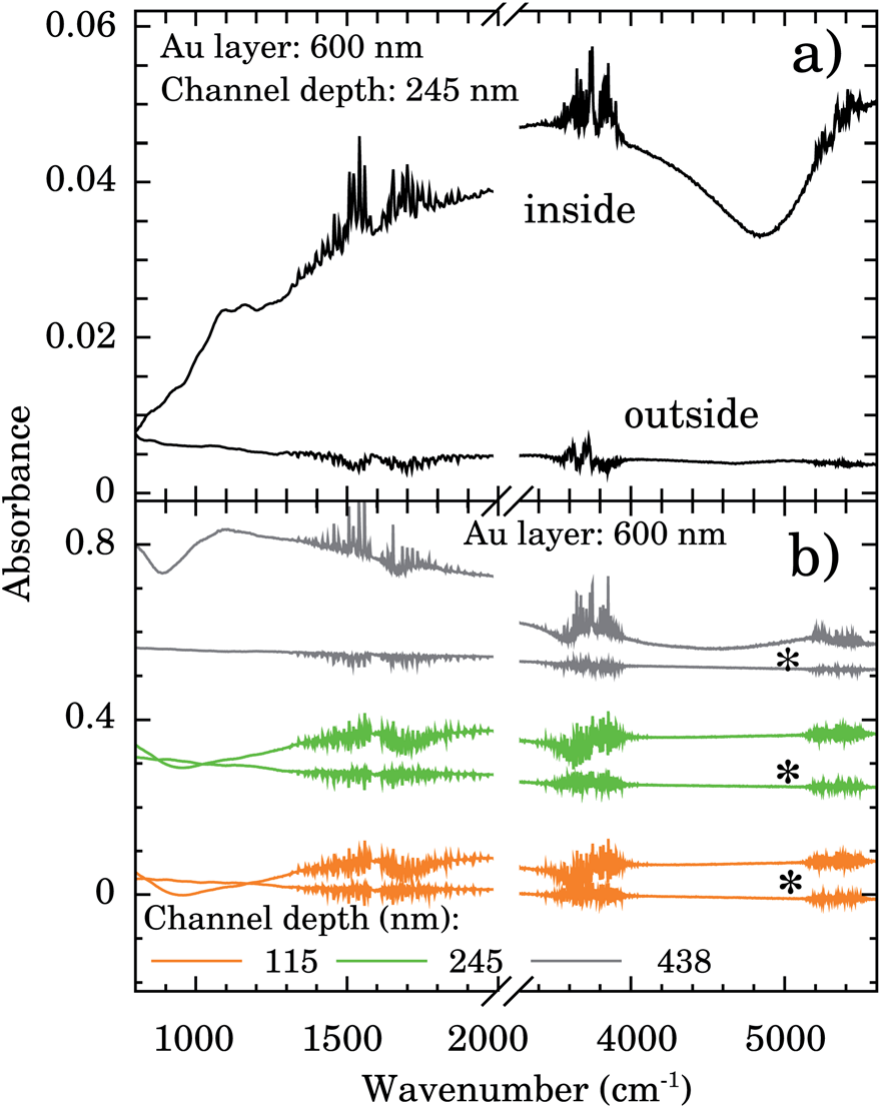
(a) FTIR spectra showing the presence of water within nanochannels of depth of 245 nm. (b) FTIR spectra showing the presence of water even after one week (*) for nanochannels of depth of 115, 245, and 438 nm on a 600 nm Au layer.

FTIR spectra were taken inside and outside the nanochannels region in order to confirm the presence of water. The spectrum inside the nanochannels region (top of Fig. 3a) presents the typical characteristic water vibrational bands.^26^ We notice that all these bands presented negligible intensities outside the channels (bottom of Fig. 3a). The broad band from 1300 to 1900 cm^−1^ refers to the bending vibration of the H_2_O molecule.^27,28^ The band from 3450 to 3950 cm^−1^ refers to the symmetrical and asymmetrical stretch vibration of the O–H bond (WAB),^28,29^ and the last band from 5150 to 5500 cm^−1^ refers to the inter-molecular hydrogen bonding vibration (WCB).^30,31^

In order to test the stability of the confinement process we checked the FTIR signal of samples after 1 week sample vacuum conditioning. Representative results are displayed in Fig. 3b for samples with nanochannels of depth of 115 nm (orange), 245 nm (green), and 438 nm (gray). The water bands are clearly detectable indicating that it is still present in the confinement region.

**Fig. 4:**
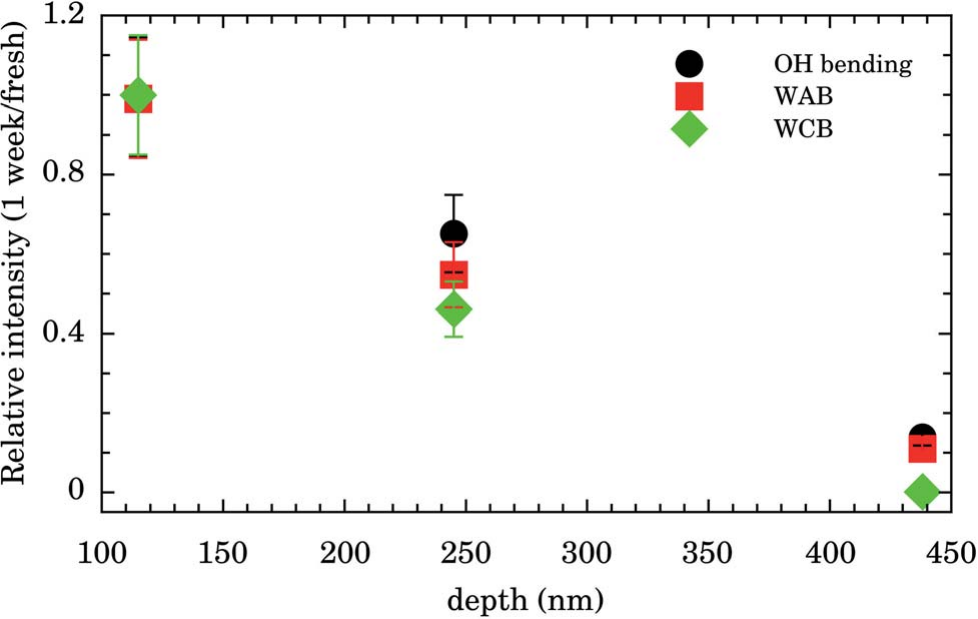
Relative integrated intensity (1 week/fresh samples intensities) of OH bending (solid black circle), symmetrical and asymmetrical stretch vibration of the O–H bond (WAB, solid red square), and inter-molecular hydrogen bonding (WCB, solid green diamond) bands.

Integrated intensities of the OH bending, WAB, and WCB bands after 1 week relative to those for fresh samples as function of nanochannels depth are shown in Fig. 4. The relative intensities (and consequently water content) for the shallowest samples of depth of 115 nm are almost constant up to 1 week. Those of intermediate values of depth (245 nm) presented an intensity decrease of ∼50% while for the deepest samples (438 nm) water band intensities after 1 week were ∼10% of the fresh ones. This finding indicates that confined water is almost bound to the Au surface for shallow samples and these presented higher hydrophilicity compared to the others. It represents important experimental evidence concerning the hydrophilicity of the first layers of wetting for Au.

**Fig. 5:**
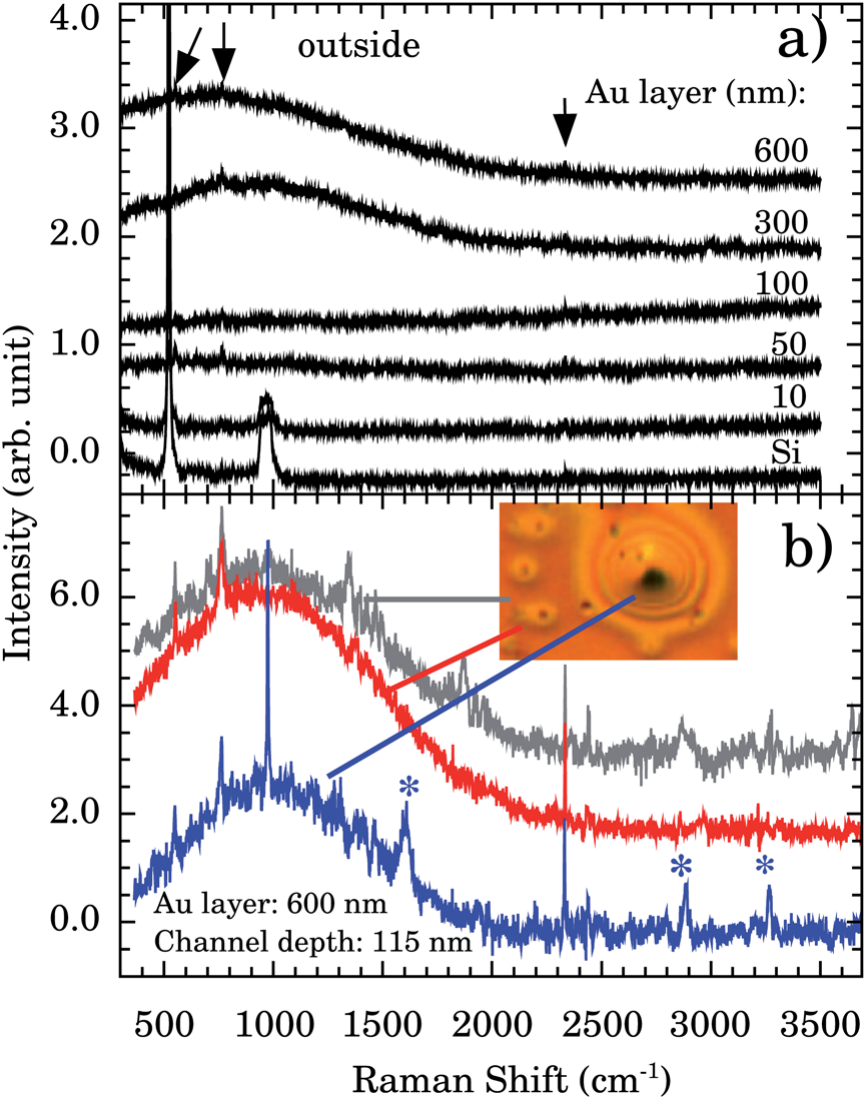
(a) Raman spectra of Au thin films (thickness of 600, 300, 100, 50, and 10 nm) deposited over n/p Si and the Si substrate. All spectra were taken outside the nanochannels. Vertical arrows indicate additional Raman bands compared to Si spectrum. (b) Representative Raman spectra taken outside (gray), around (red), and inside (blue) the observed oxidation in nanochannels of depth of 115 nm. The inset shows the optical image (100× objective) in the oxidation region. Asterisks (*) indicate the characteristic oxidation bands at ∼1602, 2890, and 3260 cm^−1^.

Fig. 5a presents the Raman spectra outside the nanochannels region for samples with Au thicknesses of 10, 50, 100, 300, and 600 nm. The Si band at ∼521 cm^−1^ and the 2^nd^ order scattering at ∼1000 cm^−1^ are observed in all samples. However, a strong and broad diffusive electronic Raman scattering for 300 and 600 nm samples is also observed. Additional tiny bands appear at 551, 765, and 2334 cm^−1^ (indicated by vertical arrows). These bands are associated with the Au–OH stretching mode,^32^ e^−^(H_2_O),^33^ and OH stretching,^34^ respectively. The presence of these bands indicates that some water molecules are adsorbed on the Au surface. This represents more evidence concerning the Au 〈111〉 first water wetting layer hydrophilicity.

We report a remarkable oxidation process around the nanochannels region (see inset of Fig. 5b). The holes observed in Fig. 5b resemble the “pitting” aspect of corrosion. It is reported that for Au 〈111〉 the surface oxidation occurs preferentially by pitting.^35,36^ We notice that the oxidation corrosion is almost Au-film thickness independent, being observed for 100, 302, and 600 nm films. Fig. 5b shows the Raman spectra inside, around, and outside of the oxidized region. Bands at ∼2890 and ∼3260 cm^−1^ are very distinctive in the oxidized region. To the best of our knowledge their assignments are not described in the literature.

**Fig. 6:**
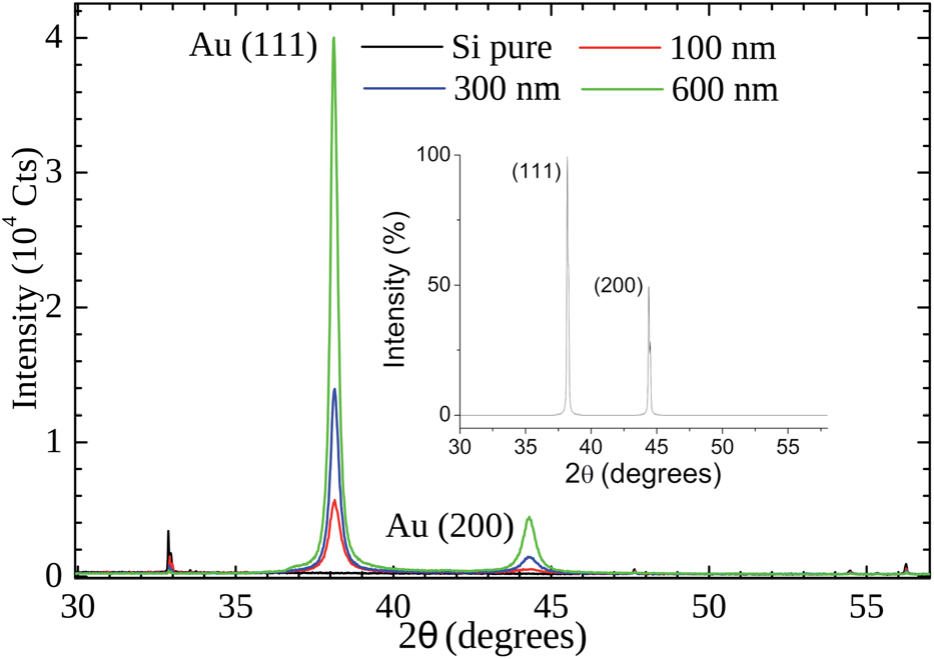
X-Ray powder diffraction patterns of gold thin films (thickness of 100 nm, 300, and 600 nm) on Si substrate. The inset shows the cubic fcc Au (PDF: 4-784) standard pattern.

Our XRD results (see Fig. 6) indicate that the (111) is the preferential orientation on our samples. Reflections from (200) crystal planes are also present but at lower intensities (∼5%). For comparison, in the fcc Au standard (see inset of Fig. 6) the (200) to (111) intensity ratio is ∼50%. A symmetrical XRD measurement (*θ*–2*θ* configuration) was performed and the relative greater intensity of the (111) reflection indicates that the crystallite faces with this orientation predominate on the samples.

The Au coupling to liquid water is still a question under debate.^37^ Recent experimental studies have shown that the surface of Au 〈111〉 at room temperature is hydrophilic and more stable than other orientations.^38^ Cicero *et al.*^39^ studied the interaction of water layers on the 〈111〉 Au surface and the issue of hydrophobicity from *ab initio* MD simulations. Their results showed that the water/Au interface is hydrophilic due to the charge transfer from oxygen to Au which favours a dynamic attractive coupling between the metal and the first adsorbed water layer. They argue that while oxygen species preferentially reside at the Au 〈111〉 sites, hydrogen atoms are evenly distributed around them. Due to the formation of “pitting”, the deposited Au film is unleavened and the potential for local nucleation occurs at the edge of the unevenness. Thus the water at these steps binds more firmly to the Au surface, with a binding energy of 105 meV for the water monomer and the surface of Au 〈111〉.^40^ Considering that each water molecule in the bilayer structure is bonded by a half Au–O bond, this means that on Au surfaces the Au–O bond contributes 12% of the total binding energy per molecule.^40^

Qiang Li *et al.*^41^ confirmed this theory of the unevenness nucleation point through simulations and previous AFM studies. They concluded that surface and edge defects determine better water adsorption on the Au substrate 〈111〉. They argued that the water clusters had initially been adsorbed to the edges of the Au steps under environmental conditions.

**Fig. 7:**
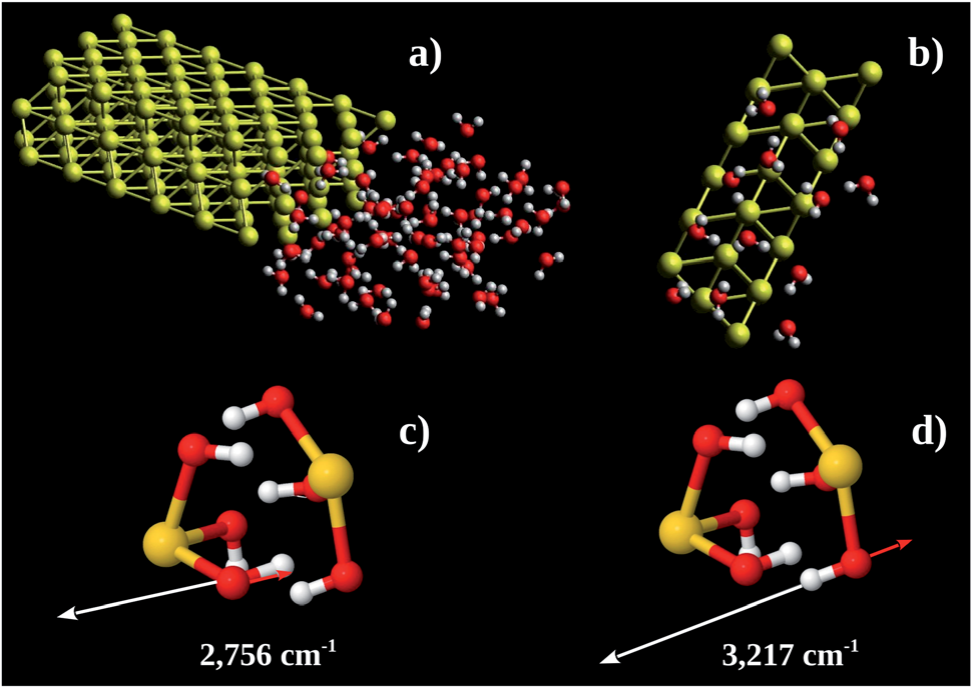
(a) Au 〈111〉 3 × 3 × 2 supercell including 67 water molecules at one side. The simulation box was 30 × 35 × 12 nm^3^. (b) Snapshot of MD showing the adsorbed water molecules around the Au 〈111〉 honeycomb face. (c and d) Eigenvectors of 2756 cm^−1^ and 3217 cm^−1^ vibrations, respectively. Yellow, red, and white spheres represent the Au, O, and H, atoms, respectively.

Using these pieces of information we tested the hypothesis of formation of some kind of Au–OH complex performing MD calculations in a Au 〈111〉 3 × 3 × 2 super-cell with one face exposed to 67 water molecules. The simulation box was 30 × 35 × 12 nm^3^. The water molecules were confined to 12 × 16 × 8 nm^3^ (see Fig. 7a). The system found equilibration after 3 fs. Fig. 7b shows a snapshot of a region close to the Au 〈111〉 surface. On average there are 6 water molecules bound to each Au 〈111〉 honeycomb (Fig. 7b). Thus the observed Au : O ratio is 1 : 3. Computing the energy of formation of 3 possible structures (Au(OH)_3_)_2_, (Au(H_2_O)_3_)_2_, and H_3_Au_3_(H_2_O)_6_, we found −302.8, −236.2 and −227.9 eV per atom, respectively. It is possible to conclude that the more stable one is (Au(OH)_3_)_2_ which corresponds to Au(OH)_3_ unit formula. We also performed vibrational calculations on (Au(OH)_3_)_2_. From these calculations we were able to assign the 2890 (calculated 2756) and 3260 (calculated 3217) cm^−1^ bands to out-of-phase (Fig. 7c) and in-phase (Fig. 7d) O–H stretching vibrations, respectively. These results enable us to argue the oxidation product observed in the Au nanochannels under confined water presence is the Au(OH)_3_ complex. This species is usually observed on supported Au-catalysts (see, *e.g.*, ref. 13 and 42).

We then performed ^1^H NMR spin-echo experiments to record the static spectra at different evolution times and to quantify the spin–spin relaxation time, aiming to describe chemical environments and the proton dynamics. However, NMR experiments in films are rarely reported since fast magic angle spinning (MAS) is difficult to implement in this geometry. Static ^1^H NMR experiments in solid state suffer from strong dipolar coupling and chemical shift anisotropy broadening which results in poorly resolved spectra, unless the species inspected are considerably mobile. As noted below, the spectra obtained are reasonably resolved and the results can be interpreted based on the extensive literature on NMR of Au nanoparticles.

**Fig. 8:**
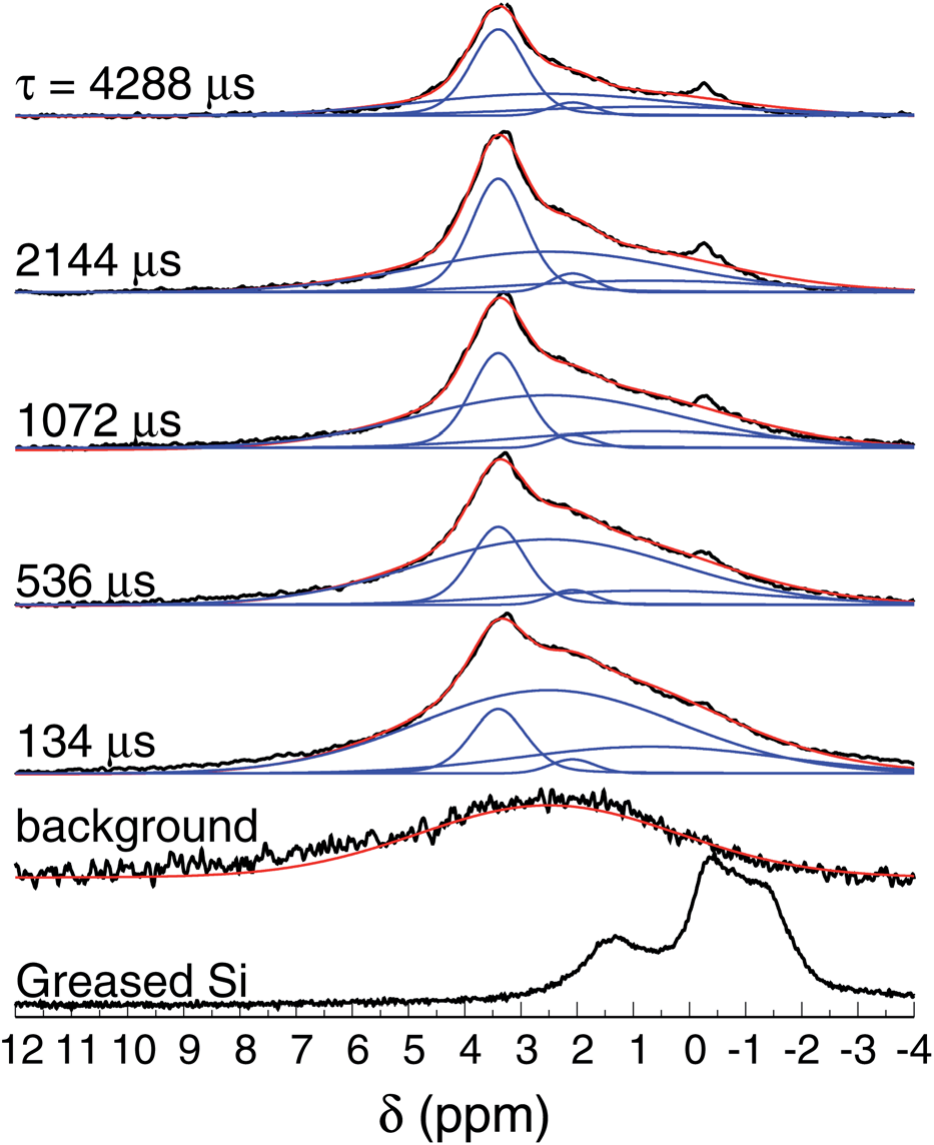
^1^H NMR spin-echo spectra of the SiAu sample (600 nm thick) as function of *τ*. The spectra of the background (empty rotor) and the Si substrate with grease are also shown.

Fig. 8 shows the normalized ^1^H spin-echo spectra of the Au film (600 nm thick) and control experiments as a function of *τ*. The ^1^H spin-echo spectra of the sample show a broad peak around 3.4 ppm. The signal of the sample is more in evidence as the evolution time of the spin-echo is set longer (*τ* > 1000 μs), as expected, since for longer evolution times the sample inside the rotor is efficiently excited while the probe-background is suppressed.

The observed spectra resemble those of amorphous solids with inhomogeneous broadening due to multiple sites and conformations. Each spectrum was carefully deconvoluted into three peaks and the background, as illustrated in Fig. 8 (see also the ESI†). The most intense peak is at 3.4 ppm (with *T*_2_ of 16 ms), there is a second peak at 2.1 ppm (*T*_2_ of 7.9 ms), and a broad peak at 0.7 ppm (*T*_2_ of 3.6 ms).

The line broadening in this case can be caused by: (i) multiple sites and conformations (distribution of chemical shift), (ii) chemical shift anisotropy, (iii) dipolar coupling, (iv) hyperfine coupling of nuclear spins to conduction electrons in the metal (Knight shift), and (v) field inhomogeneities caused by variable magnetic susceptibilities around the protons.^43–46^ The resonance shifts come from some of these features, as the chemical shift, Knight shift and the bulk magnetic susceptibility influence the effective local magnetic field.^43–46^

We noted that the measurements of the greased Si substrate and the Au covered sample show the silicone grease at the same chemical shift. This illustrates that the Au nanolayer does not contribute significantly to the overall magnetic susceptibility.^47^ Possibly, bulk magnetic susceptibility is of secondary relevance in the spectral line broadening and resonance shifts. Furthermore, the importance of the Knight shift in the overall resonance shift and line broadening in ligand grafted Au nanoparticles is long debated.^48,49^ It should be dependent, *e.g.*, on the size and shape of the Au nanolayer or the nanoparticle. More relevant, resonance shifts of the protons in the second or higher coordination to the Au on Au nanoparticles are not remarkably influenced by Knight shifts. For instance, Sharma *et al.* observed that the ^1^H NMR of triphenylphosphine (PPh_3_) is observed at 7.3 ppm as free ligand, at 7.5 ppm as Au(PPh)_3_Cl complex, and at 7.1 ppm as PPh_3_-capped gold 1.8 nm nanoparticle.^45^ For tiopronin-capped Au 0.9 nm NPs, Kohlmann *et al.* found that the methine proton shifts from 3.6 ppm to 4.3–4.7 ppm when tiopronin is attached to the Au nanoparticles.^49^ For a series of thiol-terminated-capped Au 1.7–4.2 nm NPs,^50,51^ the ^1^H downfield shifts of the grafted ligands can be as much as 1 ppm in comparison to the non-grafted ones. Thus, we can restrict our discussion considering that chemical shifts and dipolar interactions are the most important mechanisms influencing the spectral features and relaxation processes.

The first issue to be determined is to confirm the restricted motion of observed protons on the Au surface. Typically, the spin–spin relaxation rates in non-metallic solids are markedly influenced by ^1^H–^1^H homonuclear dipolar coupling and dipolar coupling to paramagnetic impurities.^52,53^ Short *T*_2_ implies strong dipolar coupling that was not averaged out by molecular motion. For instance, ^1^H *T*_2_ of water decreases from ∼1 s to 80–240 ms when confined in 50–300 μm microporous glasses.^54,55^ More closely related to our system, for triphenylphosphine capped Au nanoparticles, *T*_2_ of the protons decreases from 5.2 s in the diluted ligand to 40 ms when grafted to the 1.8 nm nanoparticle.^45^*T*_2_ of methine protons in tiopronin-capped Au 0.9–1.6 nm NPs has been reported to be around 156–55 ms.^49^ These measurements were performed in the liquid state, and unfortunately ^1^H *T*_2_ of capped Au nanoparticles in the solid state has not been reported (to the best of our knowledge). However, the shortening of *T*_2_ in the solid state is expected. Thus, we can state that protons in our case have restricted mobility, and are not adsorbed molecules on the surface.

The candidates for the signal around 3.4 ppm are confined water or stable Au–OH complexes, since adsorbed molecules do not contribute to the observed signal. In fact, the ^1^H NMR shift of adsorbed water is around 4.7–6.0 ppm,^56–58^ which is outside of the observed range. Confined water as monomer or dimer could be observed upfield shifted with respect to bulk water,^58–60^ around 0.5–1.5 ppm, and cannot be ruled out as units contributing to the NMR spectra in that shift range. Another contribution could come from Au–OH complexes, that would be observed around 4.7 ppm, taking into consideration that water as a monomer is ∼3.8 ppm upfield shifted with respect to bulk water^60^ while the Au⋯H hydrogen bond would contribute to a 3.5–4.1 ppm downfield shift (which is, obviously, a very rough additive estimation taken from quantum chemical calculations).^61^ In summary, it is possible that water monomers or dimers and Au–OH_3_ complexes could be existing species but they could only be responsible for small portions of the spectral sidebands (≈0.5–1.5 ppm and 4–6 ppm).

In order to assign the most characteristic band, centered at 3.4 ppm, we recall that for Au–OH complexes there should be electron donation from the Au to the chemisorbed molecules,^62^ similarly to Si–O bonds (to some extent). Remarkably, silanol groups in distinct configurations can be observed as a broad line from 1.8 to 4.5 ppm, the range of the shifts observed here.^59,63,64^ Furthermore, the static ^1^H NMR spectrum of MCM-41, with high concentration of surface silanol groups, is very similar to the one observed in our study.^63^ Analogously, the hydroxyl of Zr(OH)_4_ is also observed in this range.^56^ Thus, it is likely that the main observed peak around 3.4 ppm is due to protons on Au–OH complexes, in agreement with the proposed structures from quantum chemical calculations.

## 4 Conclusions

The relative intensities of water bands probed by FTIR as function of nanochannels depth (Fig. 4) and the assignment of the tiny Raman bands (Fig. 5) presented strong pieces of evidence concerning the hydrophilicity of Au 〈111〉. A unique corrosion process of the Au-nanochannels exposed to confined water was also observed. Our experimental data aimed by computer simulations indicated that the oxidation product is Au(OH)_3_, also supporting the hydrophilicity of the first wetting layer of water on Au. Moreover our findings indicate an important oxidation route of the surface which needs to be considered in Au nanosurface applications. One important example is the usage of Au in catalysis since a large amount of organic transformations in fine chemicals, pharmaceuticals, and the food industry green processes rely on Au catalytic activity (see, *e.g.*, ref. 14).

## Conflicts of interest

There are no conflicts to declare.

## Supplementary Material

RA-010-D0RA05830K-s001
