# Supplementary material for: Gold nanochannels oxidation by confined water
Source: RSC Adv. 2020 Oct 7;10(61):36980–7. doi: 10.1039/d0ra05830k (PMC9057077; doi:10.1039/d0ra05830k)
Supplement: RA-010-D0RA05830K-s001 [file RA-010-D0RA05830K-s001.pdf]

Electronic Supplementary Material (ESI) for RSC Advances.
This journal is © The Royal Society of Chemistry 2020

Supplementary Material

Gold Nanochannels Oxidation by Confined Water

André M. Batista, Thiago B. de Queiroz, Renato A. Antunes, Alexandre J. C. Lanfredi, Adriano R. V. Benvenho, Jean J. Bonvent, and Herculano Martinho

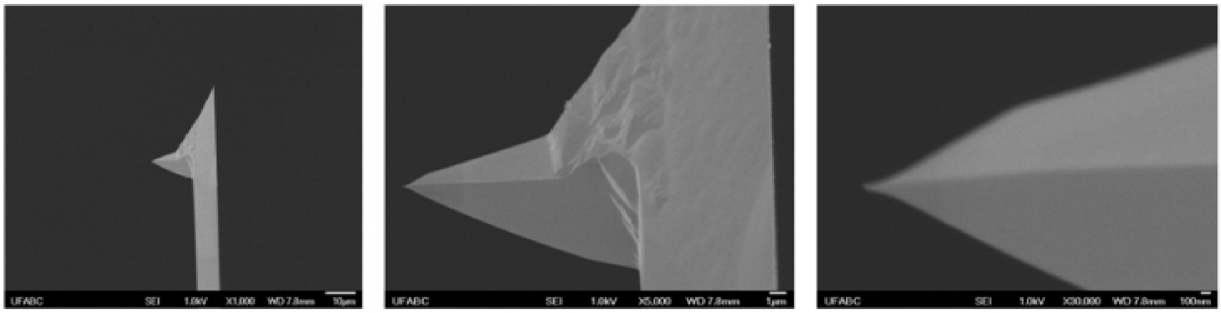

S1. AFM DLC tips SEM image.

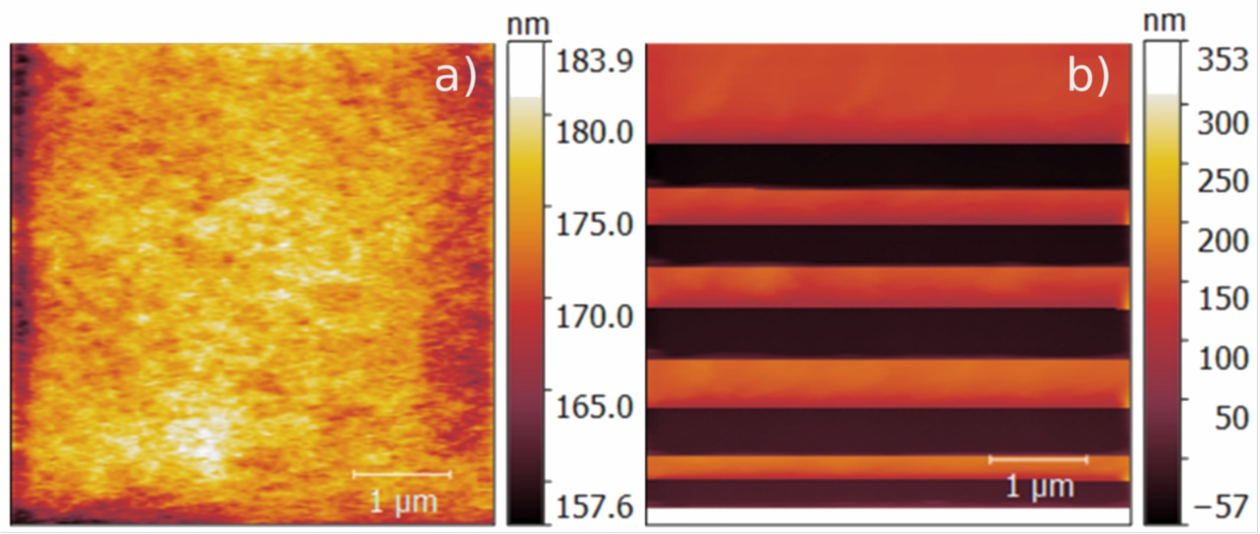

S2. AFM images for 600 nm Au layer sample taken before (a) and after (b) nanolithography.

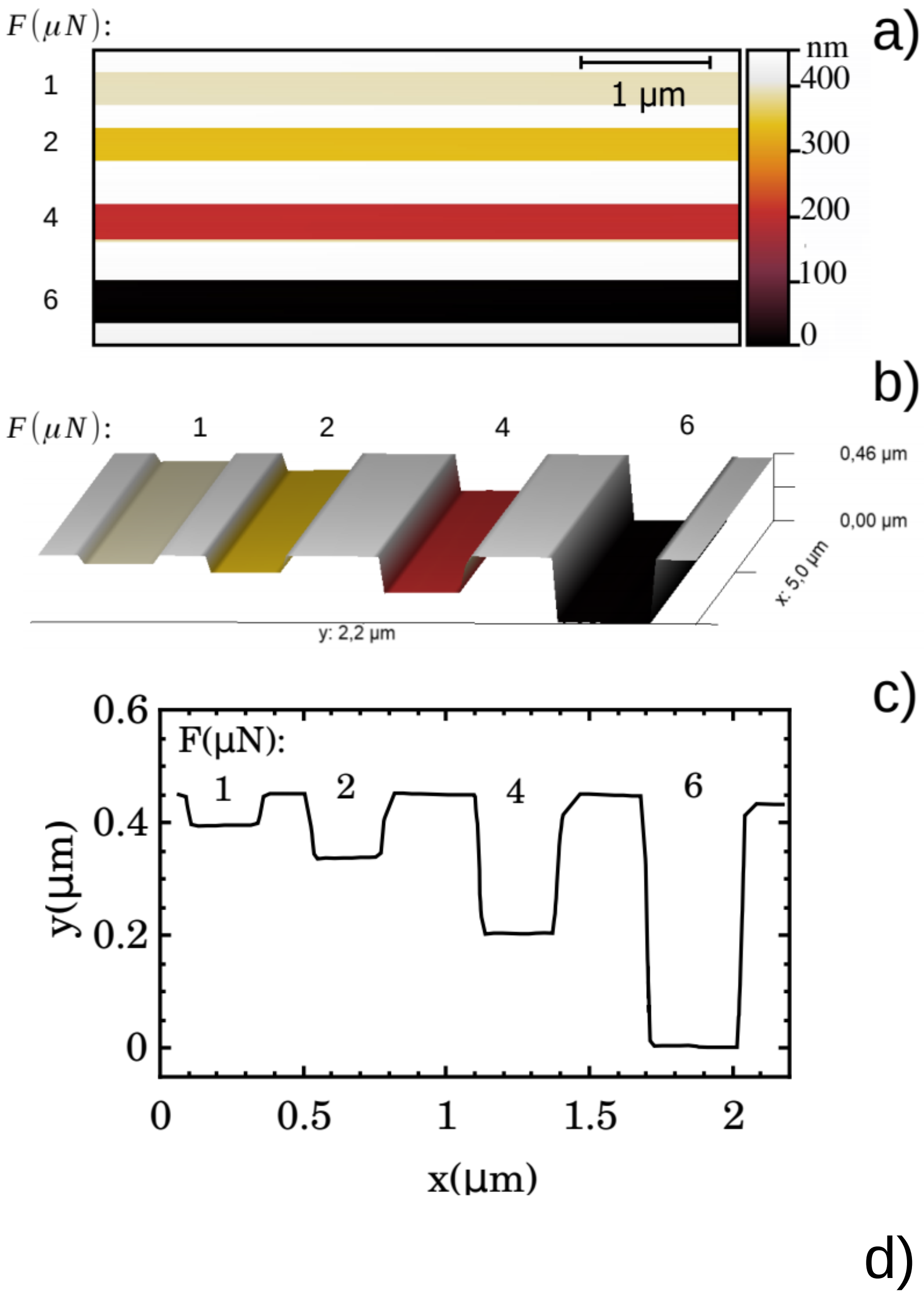

S3. Nanochannels in pseudo-color (a), 3D (b), profile plot (c) for applied forces of 1, 2, 4, and 6 μN.

NMR Data Analysis

We first deconvoluted the probe background as a Lorenztian/Gaussian distribution and included this curve with fixed parameters in the deconvolution of the spectra of the sample. Then, we deconvoluted the spectrum taken with $\tau = 4.288$ ms with additional three Gaussian/Lorentzian distributions. There are two defined peaks, at 3.4 and 2.1 ppm, while the addition of a broad peak around 0.7 was sufficient to fairly describe the spectrum. Subsequently, the other spectra are simulated as a convolution of these four distributions.

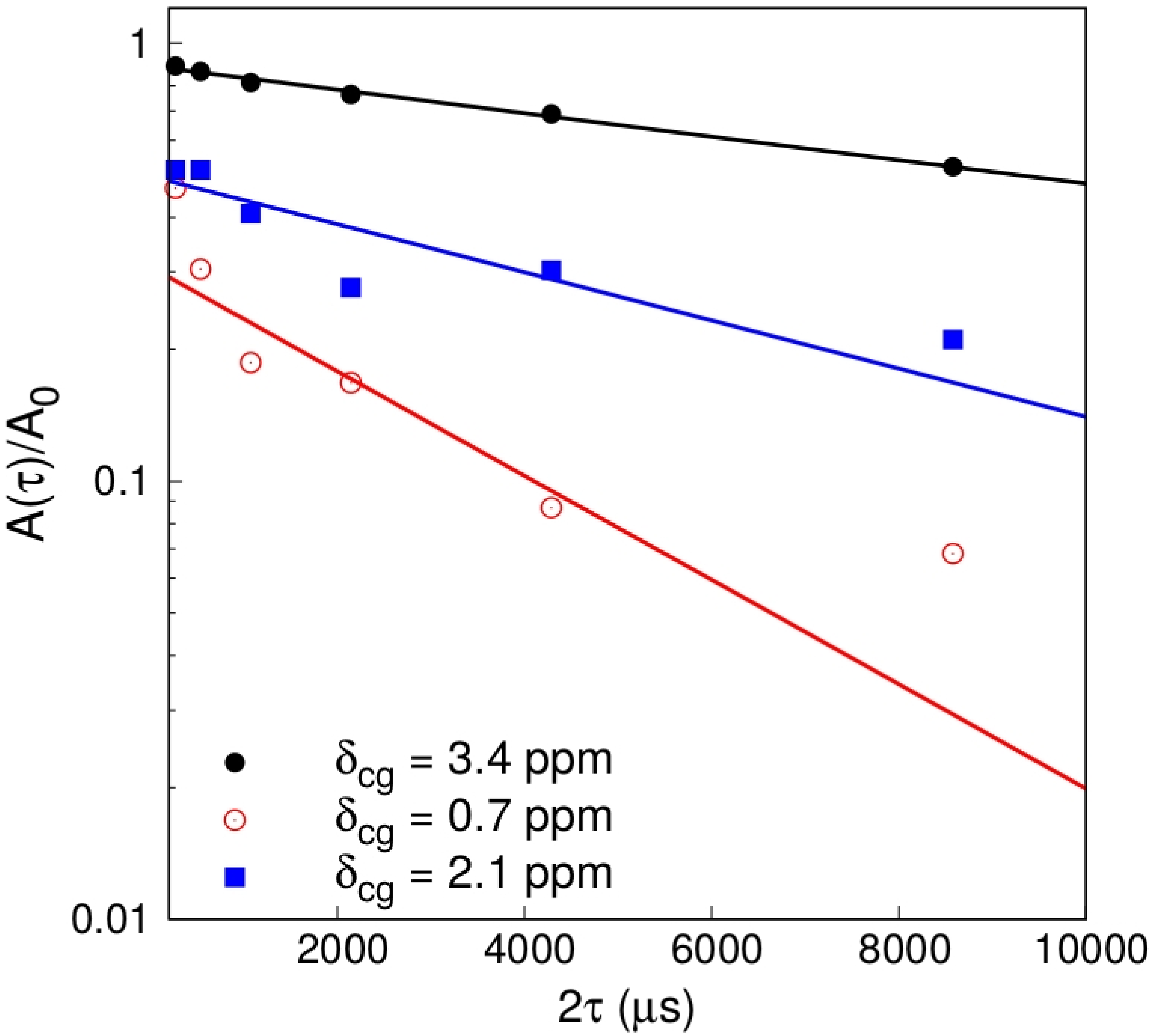

S4. Spectral intensity as a function of echo evolution time (2τ) for the deconvoluted peaks and single-exponential decay fits (lines).

Table S1. Parameters of the distributions used in the deconvolution of the spin-echo ^{1}H spectra.

δ(ppm)	Δδ(ppm)[a]	X(G,L)[b]
0.7	5.4	1
2.1	0.9	1
3.4	1.2	0.7
2.5[c]	5.4	1

a) Δδ(ppm) is the full widht at half maximum of the distribution. b) X(G, L) means the ratio between Gaussian (X) and Lorentzian (1 − X) in the curve; i.e., X = 0 stands for a full Lorentzian distribution and X = 1 for a full Gaussian distribution. c) This peaks comes from the probe background.
